# Impact of Cytochrome P450 and P-glycoprotein Gene Polymorphisms on the Risk of Hemorrhagic Complications in Older Patients with Non-Valvular Atrial Fibrillation Treated with Rivaroxaban

**DOI:** 10.3390/jpm16090470

**Published:** 2026-09-14

**Authors:** Ivan V. Sychev, Andrey P. Kondrakhin, Sherzod P. Abdullaev, Pavel O. Bochkov, Svetlana N. Tuchkova, Lyubov V. Selivanova, Denis S. Fedorinov, Olga A. Milovanova, Karin B. Mirzaev, Dmitry A. Sychev

**Affiliations:** 1 Federal State Budgetary Research Institution «Russian Research Center of Surgery Named After Academician B.V. Petrovsky», Moscow 119991, Russia; abdullaev.sp@med.ru (S.P.A.); svetlana.tuch1998@gmail.com (S.N.T.); lubovlechit711@gmail.com (L.V.S.); fedorinov.denis@gmail.com (D.S.F.); karin05doc@yandex.ru (K.B.M.); dimasychev@mail.ru (D.A.S.); 2 Moscow Healthcare Department, War Veterans Hospital No. 2, Moscow 109472, Russia; a.kondrachin@yandex.ru; 3 Federal State Budgetary Educational Institution, Ministry of Healthcare of the Russian Federation, Further Professional Education “Russian Medical Academy of Continuous Professional Education”, Moscow 125993, Russia; bok-of@yandex.ru (P.O.B.); olga_a_milovanova@mail.ru (O.A.M.); 4 Moscow Multidisciplinary Clinical Center “Kommunarka”, Moscow 108814, Russia

**Keywords:** rivaroxaban, pharmacogenetics, P-glycoprotein, *ABCB1*, hemorrhagic complications, atrial fibrillation, geriatrics, polypharmacy, drug–drug interactions

## Abstract

**Background:** The administration of direct oral anticoagulants to older adults requires careful risk stratification against a backdrop of polypharmacy and age-related renal function decline. The role of single nucleotide polymorphisms in cytochrome P450 genes and efflux transporters in this context remains a clinically understudied issue. This study aimed to investigate the association of *ABCB1*, *CYP3A4*, and *CYP3A5* allelic variants with the safety of rivaroxaban therapy in geriatric patients with non-valvular atrial fibrillation (AF). **Materials and Methods:** This prospective cohort study consecutively enrolled 94 patients (mean age 83.2 ± 9.2 years). Rivaroxaban trough plasma concentrations ($C_{min,ss}$ ) were quantified using a validated high-performance liquid chromatography-tandem mass spectrometry (HPLC-MS/MS) assay. Real-time polymerase chain reaction was utilized for the molecular genetic profiling of the *ABCB1* (rs1045642, rs2032582), CYP3A4*22 (rs35599367), and CYP3A5*3 (rs776746) loci. Comorbidity severity and the spectrum of drug–drug interactions were evaluated as additional predictors. **Results:** Clinically relevant hemorrhagic complications were documented in 36.2% (*n* = 34) of the monitored patients. A significant association was established between the homozygous CC genotype at the *ABCB1* rs1045642 locus and increased bleeding propensity (odds ratio [OR] 2.42; 95% CI: 1.02–5.74; *p* = 0.042), whereas the alternative TT variant was associated with a pronounced protective effect. No statistically significant correlations were identified for biotransformation isoenzyme markers (CYP3A4*22, CYP3A5*3). Anticoagulant metrics were comparably distributed across groups and showed no dependence on genetic status. The leading non-genetic factors associated with hemorrhage were advanced age (*p* < 0.0001), progressive glomerular filtration rate decline (CKD stages 3b–4; *p* = 0.0009), and pharmacokinetic/pharmacodynamic interference from the co-administration of amiodarone (OR 3.61), non-steroidal anti-inflammatory drugs, and antiplatelet agents. The validated HAS-BLED score demonstrated no predictive power within the comorbid conditions of this cohort (*p* = 0.052). **Conclusions:** The *ABCB1* rs1045642 (C3435T) polymorphism is associated with the occurrence of hemorrhagic events. Implementing pharmacogenetic testing of the P-glycoprotein transporter combined with a rigorous audit of renal excretory function and concomitant therapy management may optimize the safety profile of rivaroxaban in geriatric practice. These findings warrant confirmation in a larger cohort.

## 1. Introduction

Atrial fibrillation (AF) remains one of the most prevalent arrhythmias, substantially increasing the risk of cardioembolic stroke and systemic thromboembolism, particularly among older age groups [1]. According to current clinical guidelines, direct oral anticoagulants (DOACs) serve as first-line agents for preventing thromboembolic complications in non-valvular AF patients, except for clinical scenarios requiring vitamin K antagonists [2].

Prescribing anticoagulant therapy demands a strict balance between ischemic event prevention and the risk of hemorrhagic complications. Although the HAS-BLED score is traditionally applied to evaluate bleeding probability, a high calculated score does not serve as an absolute contraindication to DOAC therapy; rather, it dictates the necessity for close monitoring and the correction of modifiable risk factors [3].

In older adults, anticoagulant therapy is compounded by additional complexities due to the high frequency of comorbid pathology, polypharmacy, and age-associated declines in renal excretory function. Rivaroxaban, functioning as a direct, highly selective factor Xa inhibitor, features a convenient once-daily dosing regimen that may facilitate patient adherence to treatment [4]. The drug is metabolized predominantly via cytochrome P450 isoenzymes (CYP3A4/5 and CYP2J2) and acts as a substrate for membrane efflux transporters, primarily P-glycoprotein (P-gp)—encoded by the *ABCB1* gene—and the breast cancer resistance protein (BCRP/ABCG2) [5].

To date, the evidence base describing the influence of *ABCB1* gene polymorphisms on rivaroxaban safety is characterized by pronounced inconsistency, particularly regarding the translation of pharmacokinetic alterations into tangible clinical outcomes. The *ABCB1* gene encodes P-glycoprotein (P-gp), a membrane efflux transporter responsible for drug excretion, rather than a metabolic enzyme.

From a strictly pharmacokinetic perspective, several international studies have demonstrated a clear link between genetic markers and systemic drug exposure. The traditional functional model suggests that carrying the mutant T allele (particularly the TT genotype) at the rs1045642 locus diminishes P-gp efflux function, which theoretically leads to increased intestinal absorption and higher plasma concentrations of its substrates. Consequently, the TT genotype has been associated with higher levels of trough steady-state concentration ($C_{ss}^{min}$), dose-standardized trough concentration ($C_{ss}^{min}/D$), and peak plasma concentration ($C_{max}^{ss}$) [6,7]. A similar pharmacokinetic elevation has been recorded in carriers of the TT genotype for the rs2032582 marker [6], as well as for the minor T allele at the rs4148738 locus [8,9].

However, evidence linking these isolated pharmacokinetic shifts directly to clinically significant bleeding outcomes (such as major or clinically relevant non-major bleeding according to ISTH/BARC criteria) remains highly contradictory. The assumption that elevated plasma concentrations linearly translate into hemorrhagic events is not uniformly supported. While some data suggest that the TT genotype and associated plasma level increases correlate with prothrombin time (PT) prolongation and a rise in hemorrhagic events [7,9,10], real-world clinical observations often reveal paradoxical associations. For instance, the “wild-type” CC genotype at the rs1045642 locus has also been identified as a significant risk factor for bleeding, indicating a profound disconnection between strictly systemic pharmacokinetic parameters and ultimate clinical phenotypes.

Furthermore, actual bleeding risks are heavily modulated by drug–drug-gene interactions (phenoconversion). For example, patients undergoing concomitant treatment with potent P-glycoprotein inhibitors—such as verapamil, amiodarone, or diltiazem—exhibit a synergistic increase in hemorrhagic risk, which can effectively mask or amplify underlying genetic predispositions.

Therefore, the heterogeneity in the current literature is likely attributable to major methodological differences: fluctuations in mean age across analyzed cohorts, varying degrees of chronic kidney disease (CKD) severity, the use of mixed populations, and a critical lack of adjustments for polypharmacy [6,9]. The presence of these contradictions creates an evident gap in contemporary knowledge. It emphasizes the critical need to clearly separate systemic pharmacokinetic exposure from actual bleeding risks, evaluating these pharmacogenetic predictors within strictly stratified, comorbid geriatric cohorts where polypharmacy dictates clinical reality. Metabolic profile heterogeneity necessitates the individualization of antithrombotic therapy [7].

**Study Objective:**

To evaluate the association of the *ABCB1*, *CYP3A4*, and *CYP3A5* polymorphic markers with the risk of hemorrhagic complications in older patients with non-valvular AF receiving rivaroxaban amid polypharmacy. As a secondary endpoint, the study assessed the association between the investigated genotypes and the variability of steady-state plasma drug concentrations.

## 2. Materials and Methods

### 2.1. Study Design and Ethical Considerations

A prospective, observational, single-center cohort study was conducted. The study protocol received prior approval from the Local Ethics Committee of War Veterans Hospital No. 2, Moscow Healthcare Department (Protocol No. 226, dated 20 February 2023). All procedures strictly adhered to the ethical principles outlined in the Declaration of Helsinki of the World Medical Association and the National Standard of the Russian Federation (GOST R 52379-2005) [11]. Prior to any clinical or laboratory procedures, written informed consent was obtained from all participants. Each patient received comprehensive information detailing the study’s objectives, biomaterial sampling protocols, and potential risks.

### 2.2. Study Participants

The analytical cohort comprised patients consecutively admitted to War Veterans Hospital No. 2 between April and September 2024. The study enrolled 94 patients with confirmed non-valvular atrial fibrillation (NVAF) receiving anticoagulant therapy with rivaroxaban (15 or 20 mg once daily, tailored to clinical indications and renal function). To ensure maximum methodological rigor, the medical staff continuously monitored patient adherence to the prescribed pharmacotherapy throughout the inpatient period. This approach reliably eliminated the influence of unauthorized missed doses on the evaluated pharmacokinetic parameters and clinical outcomes. Regarding the dosing regimens within the cohort, 54 patients received the standard 20 mg/day dose, while 40 patients required a reduced 15 mg/day dose. Clinicians initiated this anticoagulant dose de-escalation strictly according to current prescribing information rather than empirically. Consistent with the overall cohort metrics, the mean age was 83.2 ± 9.2 years, with a female predominance (64%). During the observation period, hemorrhagic complications of varying severity were documented in 34 patients (36.2%).

Inclusion Criteria: Age 65 years or older; a confirmed diagnosis of NVAF; the presence of chronic kidney disease (CKD) stages 1 through 4; and signed informed consent.

Exclusion Criteria: Patients presenting with acute cardiovascular pathology at the time of screening (e.g., myocardial infarction, acute cerebrovascular accident, dissection of a major artery aneurysm) were excluded. Additional exclusion criteria included clinically significant valvular heart disease of rheumatic or other etiology, chronic heart failure stage IIb or higher, a history of cardiac surgery, alcohol-induced AF, end-stage renal disease (CKD stage 5), severe hepatic dysfunction, thyroid disorders, oncological and hematological diseases, active inflammatory processes, and acute exacerbations of gastrointestinal diseases, including gout. Patients with advanced dementia precluding independent informed consent (requiring a legal representative) were also excluded. Participants were withdrawn from the cohort if they revoked their consent or in the event of all-cause mortality.

Assessment of baseline hemorrhagic risk was performed utilizing the HAS-BLED score. The registration and classification of hemorrhagic complications occurred prospectively over the course of the index hospitalization. Consequently, the follow-up timeframe was inherently dictated by the duration of inpatient treatment, resulting in a mean observation period of 14 ± 1.9 days. Analysis of drug exposure time revealed no statistically significant differences between the cohorts with and without hemorrhagic events, which effectively mitigates the risk of time-dependent observational bias. The primary safety endpoint was defined as a composite hemorrhagic outcome, restricted to the first documented bleeding episode per patient to ensure the statistical independence of the analyzed events.

Hemorrhage severity was stratified based on the International Society on Thrombosis and Haemostasis (ISTH) consensus criteria, with events categorized into major, clinically relevant non-major (CRNM), and minor bleeding. Evaluation of the composite outcome structure (*n* = 34) demonstrated a pronounced skewness toward minor hemorrhages—predominantly recurrent epistaxis (*n* = 23)—with major bleeding accounting for only two clinical cases. This distribution dictates that the derived genetic associations primarily reflect a predisposition to minor, non-life-threatening bleeding episodes rather than massive hemorrhagic complications.

The application of an expanded composite endpoint encompassing diverse bleeding severities relies on a robust methodological rationale tailored to the geriatric profile of this real-world cohort. Against the background of advanced age, multimorbidity, and impaired glomerular filtration, even non-severe minor bleeding acquires direct clinical significance. Such episodes frequently induce anemic syndrome, require adjunctive hemostatic interventions, and serve as a primary trigger for the unwarranted premature discontinuation of direct oral anticoagulants.

Conducting a sensitivity analysis with the exclusion of minor bleeding events was a priori rejected as statistically unfeasible. Subtracting 23 minor events from the primary endpoint array would deplete the outcome count to a threshold inadequate for valid genetic association testing, inevitably causing a critical deficit in statistical power and an unacceptably high probability of a Type II error. To eliminate detection bias, the verification and expert adjudication of all bleeding events were executed by clinicians completely blinded to both the molecular genetic data and rivaroxaban plasma concentrations. Furthermore, the identification of drug–drug interactions (DDIs), with a specific focus on cytochrome P450 and P-glycoprotein inhibitors or inducers, was conducted using official prescribing information, the UpToDate Lexicomp database (Wolters Kluwer, Waltham, MA, USA; https://www.uptodate.com, accessed on 29 May 2025), and targeted FDA reference materials.

### 2.3. Molecular Genetic Analysis

Venous blood (5 mL) served as the biological matrix and was collected into standard EDTA-containing vacuum tubes (Becton Dickinson). To preserve nucleic acid integrity, the samples were initially frozen at −20 °C, followed by transport and long-term storage at −70 °C prior to genotyping.

Genomic DNA extraction utilized the column method with the QIAamp DNA Blood Mini Kit (Qiagen, Hilden, Germany). The concentration and spectrophotometric purity of the resulting DNA eluates were verified using a NanoDrop ND-1000 instrument (Thermo Fisher Scientific, Waltham, MA, USA). Genotyping was performed via real-time polymerase chain reaction (PCR) with allele-specific detection, utilizing reagent kits from Syntol (Moscow, Russia). Amplification and fluorescent signal detection were carried out on a CFX96 Touch system (Bio-Rad, Hercules, CA, USA). The protocol screened for the following polymorphic markers: *ABCB1* (rs1045642, rs2032582), CYP3A4*22 (rs35599367), and CYP3A5*3 (rs776746).

The conformity of the observed genotype frequencies to the expected values under the Hardy–Weinberg equilibrium was assessed for the entire study cohort using Pearson’s chi2-test. The actual distribution of allelic variants across all analyzed loci (ABCB1 rs1045642, ABCB1 rs2032582, CYP3A4*22, and CYP3A5*3) remained in genetic equilibrium. None of the loci exhibited statistically significant deviations from the predicted population constants (*p* > 0.05 in all cases).

### 2.4. Quantification of Rivaroxaban Plasma Concentrations

Trough steady-state plasma concentrations of rivaroxaban C__min,ss_ were measured for all enrolled patients. Blood sampling occurred strictly during pharmacokinetic steady state—defined as at least five half-lives (T__1/2_) after the initiation of a regular dosing regimen.

Venous blood was drawn into EDTA vacutainers. Centrifugation at 3000 rpm for 15 min separated the plasma, which was subsequently aliquoted into dry polypropylene Eppendorf tubes. These aliquots were stored at −70 °C until instrumental analysis.

High-performance liquid chromatography with tandem mass spectrometry (HPLC-MS/MS) quantified the rivaroxaban plasma concentrations. The analytical system comprised an Agilent 1200 liquid chromatograph (Agilent Technologies, Santa Clara, CA, USA) (equipped with a quaternary pump, vacuum mobile phase degasser, and thermostatted column compartment) coupled to an Agilent 6410 Triple Quadrupole mass spectrometer.

Analyte extraction relied on protein precipitation. Plasma samples were thawed at room temperature. A 250 µL volume of precipitation mixture (methanol and 0.1% hydrochloric acid, 9:1 *v*/*v*) was added to 100 µL of the plasma sample. After vortex mixing, the homogenate was incubated for 10 min to ensure complete macromolecular denaturation, followed by a second vortex step. Centrifugation at 10,000 rpm for 10 min yielded the supernatant, which was decanted into chromatographic vials and loaded into the autosampler.

Chromatographic separation was achieved on an Agilent Extend-C18 column (150 mm × 4.6 mm, 3.5 µm particle size) maintained at 40 °C. The mobile phase consisted of eluents A and B. Eluent A was prepared by diluting 50 mL of 0.1 M ammonium acetate and 5 mL of formic acid to 1 L with deionized water. Eluent B utilized identical volumes of ammonium acetate and formic acid, brought to 1 L with acetonitrile. An isocratic elution profile of 70:30 (A:B, *v*/*v*) was applied. The flow rate was set at 0.3 mL/min with an injection volume of 10 µL. The total run time per sample was 7 min.

Target compound detection employed multiple reaction monitoring (MRM) using positive electrospray ionization (ESI+). The ionization source operated under the following parameters: nebulizer gas (nitrogen) pressure at 35 psi, and drying gas flow at 11 L/min at 350 °C. The fragmentor voltage was set to 135 V, and the collision energy was 25 V.

### 2.5. Statistical Analysis

Statistical analyses were performed using Statistica software (v12.0; StatSoft Inc., Tulsa, OK, USA). The Kolmogorov–Smirnov test, supplemented by an evaluation of skewness and kurtosis coefficients, determined the normality of continuous variables (for n > 50). Normally distributed data are expressed as the mean and standard deviation (M ± SD). Differences between group means were evaluated using a two-sided Student’s *t*-test.

Categorical variables are summarized as absolute counts and relative frequencies. A χ^2^ goodness-of-fit test was utilized to verify whether the observed genotype distributions conformed to the Hardy–Weinberg equilibrium (HWE). Dominant and recessive inheritance models were applied to estimate genetic risk. Differences in the frequencies of categorical variables, including specific alleles and genotypes, were assessed using Pearson’s χ^2^ test. When expected frequencies in contingency table cells were insufficient, Fisher’s exact test was employed.

The strength of the association between the targeted genetic markers and the occurrence of hemorrhagic events was quantified by calculating the odds ratio (OR) alongside a 95% confidence interval (95% CI). Across all statistical procedures, the null hypothesis was rejected at a significance level of *p* < 0.05.

## 3. Results

The overall incidence of hemorrhagic complications within the study cohort was 36.2% (*n* = 34). Based on ISTH criteria, the vast majority of these episodes were classified as clinically relevant non-major (CRNM) or minor bleeding. Non-severe events predominantly consisted of epistaxis (*n* = 23), followed by macrohematuria (*n* = 4), ecchymosis (n = 4), and gastrointestinal bleeding (*n* = 1). Major, life-threatening bleeding occurred in two patients.

### 3.1. Clinical, Demographic, and Laboratory Characteristics

Comparative analysis revealed that patients who developed hemorrhagic complications were significantly older. The mean age in the bleeding subgroup was 88.8 ± 8.4 years, compared with 80.1 ± 8.7 years in the event-free group (p<0.0001). Although patients with complications exhibited a trend toward lower anthropometric values—with a mean body weight of 74.4 ± 15.4 kg versus 79.1 ± 16.7 kg in controls—this difference did not reach statistical significance (p=0.195). Body mass index (BMI) was similarly distributed between the cohorts, measuring 28.1 ± 5.3 kg/m^2^ in the bleeding group and 29.0 ± 5.8 kg/m^2^ in the complication-free group (p=0.472).

Baseline clinical assessment underscored the multimorbidity of this geriatric cohort. Every enrolled patient (100%) had a documented history of arterial hypertension and chronic heart failure (CHF). The prevalence of other comorbid conditions, such as type 2 diabetes mellitus and prior acute cerebrovascular events (stroke or transient ischemic attack), was comparable across subgroups and showed no statistically significant association with the occurrence of hemorrhage (p>0.05).

Evaluation of baseline hemorrhagic risk using the HAS-BLED score yielded a mean of 5.7 ± 1.1 for the entire study cohort, stratifying all enrolled patients into a high-risk category. Comparative analysis determined that the mean calculated scores did not differ significantly between the cohort with documented clinical bleeding and the control group (5.4 ± 1.3 vs. 5.9 ± 0.9, respectively; p=0.052).

Peripheral blood parameters did not differ significantly between the cohorts. Hemoglobin (114.9 ± 18.7 vs. 122.6 ± 19.5 g/L; *p* = 0.072) and erythrocyte counts (4.05 ± 0.75 vs. 4.24 ± 0.62 × 10^12^/L; *p* = 0.223) exhibited only a marginal downward trend in patients with complications. Similarly, the slight reduction in platelet count observed in the bleeding group (214.9 ± 61.3 vs. 229.2 ± 72.7 × 10^9^/L) lacked statistical significance (*p* = 0.343).

### 3.2. Analysis of Concomitant Pharmacotherapy and Drug–Drug Interactions

Given the high comorbidity burden within the geriatric cohort, patients were frequently exposed to pronounced polypharmacy. We evaluated concomitant therapy to identify clinically relevant drug–drug interactions capable of altering the safety profile of rivaroxaban. Documented interactions were categorized as either pharmacokinetic (affecting anticoagulant metabolism and excretion) or pharmacodynamic (exerting synergistic effects on hemostasis) (Table 1).

#### 3.2.1. Pharmacokinetic Interactions

Because rivaroxaban acts as a substrate for both the CYP3A4 isoenzyme and the P-glycoprotein (P-gp) efflux transporter, co-administration with inhibitors of these pathways can reduce drug clearance and elevate plasma concentrations. Within the analyzed cohort, the moderate P-gp/CYP3A4 inhibitor amiodarone demonstrated a clinically notable association. Concomitant amiodarone use correlated with a higher bleeding frequency, as complications occurred in 20 of the 37 patients receiving this agent. Odds ratio analysis indicated that amiodarone therapy was associated with a 3.6-fold increase in bleeding probability (OR 3.61; 95% CI: 1.49–8.75; *p* = 0.004).

Non-dihydropyridine calcium channel blockers (verapamil and diltiazem) and cardiac glycosides (digoxin) exhibited only a weak trend toward increased risk (OR 1.69 and OR 1.80, respectively). These values did not reach statistical significance (*p* > 0.05), potentially reflecting a milder inhibitory profile compared to amiodarone.

#### 3.2.2. Pharmacodynamic Interactions

A second category of medications significantly associated with hemorrhagic events included agents that directly modulate the coagulation cascade or platelet function. In this cohort, combining rivaroxaban with antiplatelet agents (predominantly acetylsalicylic acid) was associated with a 3-fold increase in the odds of major and clinically relevant non-major bleeding (OR 3.00; 95% CI: 1.23–7.31; *p* = 0.014).

The concurrent use of non-steroidal anti-inflammatory drugs (NSAIDs), such as diclofenac and ketorolac, served as an additional contributing factor. The inhibition of thromboxane-dependent platelet aggregation, coupled with the direct ulcerogenic effect of NSAIDs on the gastrointestinal mucosa, likely facilitated these complications, yielding a 3.2-fold higher bleeding probability (OR 3.21; 95% CI: 1.29–7.98; *p* = 0.009).

Other medications in the evaluated regimen that lack clinically significant pharmacological overlap with rivaroxaban (e.g., beta-blockers, statins) expectedly demonstrated no statistically significant association with bleeding frequency (*p* = 0.178 and *p* = 0.207, respectively).

### 3.3. Assessment of Renal Function and Hematological Parameters

Renal excretory function was evaluated using serum urea and creatinine levels, alongside the estimated glomerular filtration rate (eGFR). Patients who experienced hemorrhagic events exhibited a clinically pronounced and statistically significant reduction in eGFR (38.6 ± 16.4 vs. 57.8 ± 21.0 mL/min/1.73 m^2^; *p* < 0.0001). This subgroup also displayed a consistent trend toward azotemia. Urea concentrations reached 9.35 ± 4.36 mmol/L (compared to 7.83 ± 3.24 mmol/L; *p* = 0.079), while creatinine levels measured 114.1 ± 34.1 µmol/L (vs. 101.9 ± 29.0 µmol/L; *p* = 0.087).

Stratifying the cohort by chronic kidney disease (CKD) stage confirmed that progressive renal impairment is strongly associated with complications during rivaroxaban therapy (Table 2). The odds of bleeding escalated significantly starting at CKD stage 3b (OR 2.33; 95% CI: 1.09–4.98; *p* = 0.028) and peaked among patients presenting with CKD stage 4 (OR 4.09; 95% CI: 1.73–9.70; *p* = 0.0009).

Evaluating the broader clinical profile indicates that advanced age and compromised renal excretory function are the principal non-genetic factors associated with bleeding frequency in this cohort of rivaroxaban-treated patients. Routine peripheral blood parameters failed to demonstrate utility as reliable independent predictors of hemorrhage within this specific sample.

### 3.4. Pharmacogenetic Testing Results

The distribution of allelic variants and genotypes across the targeted polymorphic loci is presented in Table 3. The analysis revealed a statistically significant association between the homozygous CC genotype of the *ABCB1* rs1045642 (C3435T) polymorphism and bleeding risk (OR 2.42; 95% CI: 1.02–5.74; *p* = 0.042). Under a recessive inheritance model for the C allele (CC versus CT + TT), the odds ratio was 2.42 (*p* = 0.042). A dominant model (CC + CT versus TT homozygotes) yielded an OR of 4.08 (95% CI: 1.11–14.94; *p* = 0.019). It must be noted, however, that the TT genotype was relatively rare within the studied cohort, which is reflected in the wide confidence intervals.

Evaluations of the *ABCB1* rs2032582, CYP3A5*3 (rs776746), and CYP3A4*22 (rs35599367) loci yielded no statistically significant frequency differences between the bleeding and non-bleeding cohorts (*p* > 0.05). The heterozygous GA genotype at the *CYP3A5* locus displayed a numerical trend toward increased risk (OR 2.00), though the wide confidence interval (0.67–5.92; *p* = 0.200) precludes confirming statistical significance in the current sample size.

### 3.5. Assessment of Rivaroxaban Pharmacokinetics

As a secondary endpoint, we evaluated the association of genetic polymorphisms and concomitant clinical factors with the trough steady-state plasma concentration (C_min,ss) of rivaroxaban. Comparative analysis revealed no statistically significant differences in plasma drug concentrations between the subgroup with documented bleeding events and the complication-free control cohort (*p* > 0.05). Absolute anticoagulant concentrations in both groups exhibited pronounced interindividual variability. This pattern is entirely expected for a geriatric cohort presenting with diverse comorbidities and varying degrees of renal impairment. Stratifying the cohort based on pharmacogenetic profiles yielded similar outcomes. Comparing carriers of different allelic variants across the ABCB1 (rs1045642, rs2032582), CYP3A4*22, and CYP3A5*3 loci revealed no significant divergence in rivaroxaban plasma concentrations (*p* > 0.05 for all comparisons).

Notably, patients with the homozygous CC genotype at the ABCB1 rs1045642 locus—who exhibited a clinically significant 2.4-fold increase in bleeding risk—maintained systemic drug concentrations that were statistically indistinguishable from those observed in carriers of the alternative CT and TT genotypes. This negative pharmacokinetic finding holds independent clinical value. The absence of a direct linear correlation suggests a highly multifaceted pathogenesis for hemorrhage in older adults. Shifts in the tissue distribution of the drug do not necessarily translate directly into altered systemic plasma exposure (C_min,ss) within the venous circulation. Furthermore, this outcome reinforces the dominant role of clinical factors—specifically CKD and drug–drug interactions—in modifying bleeding vulnerability. Such clinical variables can critically destabilize the hemostatic system even when rivaroxaban plasma concentrations fall within expected on-treatment ranges.

## 4. Discussion

The overall incidence of hemorrhagic complications in our study reached 36.2%. While this figure initially appears substantially higher than the rates reported in pivotal randomized clinical trials (e.g., ROCKET AF) [10], it aligns perfectly with recent real-world evidence focusing on extremely old patient cohorts [12]. This elevated event rate stems from the prospective study design, which mandated the rigorous documentation of all clinically relevant non-major (CRNM) and minor bleeding episodes, alongside major events. Additional aggravating factors included the cohort’s geriatric profile (mean age in the complication subgroup reached 88.8 years), a high prevalence of severe chronic kidney disease (CKD stages 3b–4), and the widespread, necessary use of ulcerogenic medications and concomitant antiplatelet therapy.

Assessing traditional hemorrhagic risk stratification tools in this specific population deserves distinct consideration. In our study, mean HAS-BLED scores did not differ significantly between patients who developed bleeding and the control cohort. The observed numerical trend—where the complication-free group exhibited slightly higher absolute values—most likely reflects the pronounced clinical homogeneity of the sample. The advanced age of the patients, combined with a universally high burden of comorbid pathology, shifted the entire cohort into a maximum-risk category (mean score 5.7).

The present study sought to investigate the association of polymorphic variants in genes encoding the P-glycoprotein membrane transporter (ABCB1) and key cytochrome P450 enzymes (CYP3A4, CYP3A5) with the cumulative risk of hemorrhagic complications in older patients receiving rivaroxaban for non-valvular atrial fibrillation (NVAF). Given the participants’ geriatric status and multiple intercurrent pathologies, we analyzed the anticoagulant’s biotransformation pathways within a context of pronounced polypharmacy.

The primary finding of the molecular genetic analysis was a statistically significant association between the homozygous CC genotype at the ABCB1 rs1045642 locus and the occurrence of bleeding (OR 2.42 under a recessive inheritance model). It is of particular scientific interest that this elevated hemorrhagic risk correlates precisely with the “wild-type” (functionally normal) genotype. According to classical pharmacokinetics, the presence of the C allele dictates higher P-glycoprotein expression. Theoretically, this should limit rivaroxaban absorption in enterocytes and accelerate its renal excretion. However, the global literature presents highly conflicting data regarding the impact of ABCB1 polymorphisms on the safety profile of direct oral anticoagulants (DOACs) [13].

Our findings demonstrate a paradoxical association where the complex, tissue-specific, and organotropic functional profile of the homozygous ABCB1 CC genotype corresponds with an increased risk of hemorrhagic events. This observation contradicts classical pharmacokinetic models, which presume that the C allele determines higher P-glycoprotein efflux activity.

To potentially explain this phenomenon, one might hypothesize an altered local tissue distribution of the anticoagulant, specifically at the blood–brain barrier and within the microcirculatory endothelium. Nevertheless, this concept must be treated strictly as a speculative hypothesis. Because the current methodology was confined to quantifying the drug exclusively in the systemic venous circulation, verifying such tissue-specific pharmacokinetic shifts would necessitate independent experimental studies utilizing appropriate tissue models.

Additionally, under conditions of severely reduced glomerular filtration (CKD stages 3b–4)—which characterized the vast majority of patients in the complication group—organic nephron damage rigidly limits the renal clearance of rivaroxaban [14]. Such impairment completely negates any potential protective effect that high P-glycoprotein activity might exert on systemic excretion, while preserving its modulating influence on local tissue distribution.

Carrying the other investigated genetic markers—ABCB1 rs2032582 (G2677T), CYP3A5*3 (rs776746), and CYP3A4*22 (rs35599367)—demonstrated no statistically significant link to bleeding frequency. Rivaroxaban is metabolized predominantly via the CYP3A4 isoenzyme. The CYP3A4*22 allelic variant is known to induce alternative splicing, causing a 40–50% reduction in enzymatic activity [15]. However, the low population frequency of this minor allele among individuals of European descent (in our cohort, CT heterozygotes accounted for only 16% of cases, and TT homozygotes were entirely absent) precluded achieving the statistical power necessary to detect reliable differences. Regarding the non-functional CYP3A5*3 allele, which has a prevalence exceeding 85% in European populations, the lack of a clinically relevant association is explained by a nearly complete, genetically determined absence of functional CYP3A5 protein across the population. Consequently, the CYP3A4 isoenzyme entirely compensates for its metabolic role.

Beyond genetic determinants, this study identified an association between non-genetic clinical predictors and drug accumulation. Advanced age emerged as one of the most prominent risk factors tied to complications: the mean age was 88.8 years in the bleeding group versus 80.1 years in the control group. Reduced renal excretory function also correlated with complication risk. The estimated glomerular filtration rate (eGFR) was significantly lower in patients who experienced bleeding (38.6 vs. 57.8 mL/min/1.73 m^2^). Cohort stratification revealed that the probability of hemorrhage escalates significantly starting at CKD stage 3b (OR 2.33) and peaks at CKD stage 4 (OR 4.09). Because approximately 35% of administered rivaroxaban is eliminated unchanged by the kidneys, progressive declines in renal clearance inevitably drive systemic anticoagulant accumulation [14].

The analysis of concomitant drug–drug interactions (DDIs) merits separate consideration. Co-administering rivaroxaban with amiodarone—a moderate P-gp/CYP3A4 inhibitor—was associated with an increased bleeding risk, aligning with previous studies [16]. Pharmacodynamic interference from the concurrent use of antiplatelet agents (acetylsalicylic acid) and non-steroidal anti-inflammatory drugs (NSAIDs) elevated the likelihood of complications by 3.0- and 3.2-fold, respectively. Concurrently, the trough steady-state concentration of rivaroxaban did not differ statistically between the cohorts, nor did it correlate with genotypes. This observation points toward a complex, multifactorial etiology for hemorrhagic complications in older adults.

This study possesses several substantial methodological limitations that must be considered when interpreting the data. First, the single-center design, the restricted sample size (*n* = 94), and the small absolute number of recorded bleeding episodes precluded the development of a validated multivariable prognostic model. This constraint maintains a high risk of unmeasured clinical confounders influencing the final associations. Second, post hoc analysis confirmed that the assembled cohort possessed strictly limited statistical power to detect the clinical effects of rare allelic variants (such as CYP3A4*22). Third, given the hypothesis-generating nature of the study, statistical corrections for multiple comparisons were intentionally omitted to minimize the risk of a Type II error. Consequently, the identified genetic correlations remain strictly exploratory and carry an elevated probability of a Type I error. Furthermore, the composite endpoint utilized in the protocol exhibited marked heterogeneity regarding the severity of hemorrhagic events, and the study lacks an independent cohort for external validation. Finally, the current dataset does not support a definitive conclusion that integrating ABCB1 pharmacogenetic testing meaningfully improves hemorrhagic risk prediction beyond the capabilities of standard clinical assessment scores.

## 5. Conclusions

The ABCB1 rs1045642 (C3435T) polymorphism, which encodes the P-glycoprotein efflux transporter, emerged as a potentially relevant candidate pharmacogenetic biomarker associated with the safety profile of anticoagulant therapy in older adults. Carrying the homozygous CC genotype corresponded with a 2.4-fold increase in the odds of developing hemorrhagic complications during rivaroxaban treatment. Regarding the polymorphic markers for cytochrome P450 isoenzymes (CYP3A5*3 and CYP3A4*22), no statistically significant link to bleeding frequency was observed within the analyzed geriatric cohort. Given the limited statistical power of the present study to detect rare allelic variants, these findings remain exploratory. They do not definitively rule out the possibility that these markers exert a weak, modulating clinical influence.

Alongside genetic traits, the analysis identified clear associations with non-genetic clinical variables. These included advanced age (exceeding 80 years), a progressive decline in glomerular filtration rate (CKD stages 3b and 4), and pronounced polypharmacy. Pharmacokinetic and pharmacodynamic drug–drug interactions also exhibited notable clinical relevance, particularly those involving P-glycoprotein inhibitors (amiodarone) and the concurrent use of hemostasis-modulating medications (antiplatelet agents and non-steroidal anti-inflammatory drugs).

Ultimately, the observed association between the ABCB1 rs1045642 locus and bleeding risk constitutes an important exploratory pharmacogenetic signal.

## Figures and Tables

**Table 1:** Clinical, demographic, and laboratory characteristics of the study cohort stratified by the occurrence of hemorrhagic complications.

Parameter	Total Cohort (*n* = 94)	No Bleeding (*n* = 60)	Bleeding (*n* = 34)	Test Statistic (t/χ^2^)	*p*-Value
Clinical and Demographic Parameters					
Age, years	83.2 ± 9.2	80.1 ± 8.7	88.8 ± 8.4	t = −4.74 *	<0.0001
Body mass index (BMI), kg/m^2^	28.7 ± 5.6	29.0 ± 5.8	28.1 ± 5.3	t = 0.72 *	0.472
eGFR (CKD-EPI), mL/min/1.73 m^2^	49.7 ± 21.1	57.8 ± 21.0	38.6 ± 16.4	t = 4.59 *	<0.0001
HAS-BLED score, mean	5.7 ± 1.1	5.9 ± 0.9	5.4 ± 1.3	t = 1.99 **	0.052
Comorbidities, *n* (%)					
Arterial hypertension	94 (100%)	60 (100%)	34 (100%)	χ^2^ = 0.00	1.000
Chronic heart failure	94 (100%)	60 (100%)	34 (100%)	χ^2^ = 0.00	1.000
Type 2 diabetes mellitus	14 (14.9%)	9 (15.0%)	5 (14.7%)	χ^2^ = 0.002	0.967
History of stroke/TIA	7 (7.4%)	5 (8.3%)	2 (5.9%)	χ^2^ = 0.191	0.697 ***
Concomitant Medications, *n* (%)					
Amiodarone	37 (39.3%)	17 (28.3%)	20 (58.8%)	χ^2^ = 8.44	0.004
Verapamil/Diltiazem	15 (15.9%)	8 (13.3%)	7 (20.5%)	χ^2^ = 0.84	0.359
Antiplatelet agents (e.g., Aspirin)	32 (34.0%)	15 (25.0%)	17 (50.0%)	χ^2^ = 6.04	0.014
NSAIDs (e.g., Diclofenac, Ketorolac)	29 (30.8%)	13 (21.6%)	16 (47.0%)	χ^2^ = 6.75	0.009
Beta-blockers	66 (70.2%)	45 (75.0%)	21 (61.7%)	χ^2^ = 1.81	0.178
Statins	78 (82.9%)	52 (86.6%)	26 (76.4%)	χ^2^ = 1.59	0.207

* Student’s *t*-test was utilized for continuous variables exhibiting a normal distribution. ** Welch’s *t*-test was applied due to the inequality of variances. *** The *p*-value was calculated using Fisher’s exact test (two-tailed).

**Table 2:** Distribution of patients by chronic kidney disease (CKD) stage and the associated risk of hemorrhagic complications.

CKD Stage	eGFR (mL/min/1.73 m^2^)	Total Patients (*n* = 88) *	No Bleeding (*n* = 55)	Bleeding (*n* = 33)	χ^2^	*p*-Value	Odds Ratio, OR (95% CI)
**CKD Stage 2**	60–89	14	12	2	0.99	0.320	0.51 (0.13–1.96)
**CKD Stage 3a**	45–59	18	14	4	0.21	0.650	0.78 (0.26–2.33)
**CKD Stage 3b**	30–44	30	18	12	4.82	0.028	2.33 (1.09–4.98)
**CKD Stage 4**	15–29	26	11	15	11.08	0.0009	4.09 (1.73–9.70)
**Total**	-	88	55	33	17.10	0.0007	-

* Six patients lacking evidence of CKD (eGFR ≥ 90 mL/min/1.73 m^2^) were excluded from this analysis.

**Table 3:** Genotype frequency distribution and hemorrhagic risk assessment in patients receiving rivaroxaban therapy.

Gene/Polymorphism	Genotype	Bleeding (*n* = 34) *, *n* (%)	No Bleeding (*n* = 60) *, *n* (%)	χ^2^	*p*-Value	Odds Ratio, OR (95% CI)
**ABCB1 rs1045642 (C3435T)**	**CC**	21 (61.8%)	24 (40.0%)	4.12	0.042	2.42 (1.02–5.74)
	**CT**	10 (29.4%)	19 (31.7%)	0.05	0.820	0.90 (0.36–2.25)
	**TT**	3 (8.8%)	17 (28.3%)	4.93	0.026	0.25 (0.07–0.91)
**ABCB1 rs2032582 (G2677T)**	**GG**	15 (44.1%)	22 (36.7%)	0.51	0.470	1.36 (0.58–3.21)
	**GT**	13 (38.2%)	26 (43.3%)	0.23	0.630	0.81 (0.34–1.91)
	**TT**	6 (17.6%)	12 (20.0%)	0.08	0.780	0.86 (0.29–2.53)
CYP3A5*3 (rs776746)	**GG**	26 (76.5%)	52 (86.7%)	1.60	0.200	0.50 (0.17–1.48)
	**GA**	8 (23.5%)	8 (13.3%)	1.60	0.200	2.00 (0.67–5.92)
CYP3A4*22 (rs35599367)	**CC**	27 (84.4%)	50 (83.3%)	0.02	0.890	1.08 (0.33–3.48)
	**CT**	5 (15.6%)	10 (16.7%)	0.02	0.890	0.93 (0.28–2.99)

* Percentages were calculated based on the total number of patients within the respective subgroup (Bleeding/No Bleeding). The missing data for the CYP3A4*22 polymorphic locus in the bleeding subgroup (*n* = 32 instead of 34) resulted from the exclusion of two biological samples from the final analysis due to technical artifacts occurring during the PCR amplification step.

## Data Availability

The datasets generated and analyzed during this study are not publicly available due to ethical restrictions and patient confidentiality protections under the Russian Federation laws on personal data protection (Federal Law No. 152-FZ). However, anonymized data supporting the findings may be made available upon reasonable request from qualified researchers, subject to approval by the Local Ethics Committee of the War Veterans Hospital No. 2 of the Moscow Healthcare Department (contact: gvv2@zdrav.mos.ru). Requests should include a detailed research proposal and data protection plan.

## Abbreviations

The following abbreviations are used in this manuscript:

ABCB1	ATP-binding cassette subfamily B member 1
ABCG2	ATP-binding cassette subfamily G member 2
AF	Atrial fibrillation
BCRP	Breast cancer resistance protein
BMI	Body mass index
CHF	Chronic heart failure
CKD	Chronic kidney disease
CKD-EPI	Chronic Kidney Disease Epidemiology Collaboration
CRNM	Clinically relevant non-major bleeding
CYP	Cytochrome P450
DDI	Drug–drug interaction
DOAC	Direct oral anticoagulant
eGFR	Estimated glomerular filtration rate
ESI+	Positive electrospray ionization
HPLC-MS/MS	High-performance liquid chromatography-tandem mass spectrometry
HWE	Hardy–Weinberg equilibrium
INR	International normalized ratio
ISTH	International Society on Thrombosis and Haemostasis
MRM	Multiple reaction monitoring
NSAID	Non-steroidal anti-inflammatory drug
NVAF	Non-valvular atrial fibrillation
P-gp	P-glycoprotein
PCR	Polymerase chain reaction
TIA	Transient ischemic attack
